# Research progress on effects of traditional Chinese medicine on myocardial ischemia–reperfusion injury: A review

**DOI:** 10.3389/fphar.2022.1055248

**Published:** 2022-12-06

**Authors:** Na Xing, Xiao-Tong Long, Hui-Juan Zhang, Li-Dan Fu, Jian-Yuan Huang, Abdallah Iddy Chaurembo, Francis Chanda, Yun-Jing Xu, Chi Shu, Kai-Xuan Lin, Ke Yang, Han-Bin Lin

**Affiliations:** ^1^ Zhongshan Institute for Drug Discovery, SIMM CAS, Zhongshan, Guangdong, China; ^2^ Shanghai Institute of Materia Medica, Chinese Academy of Sciences, Shanghai, China; ^3^ Department of Physiology, University of Toronto, Toronto, ON, Canada; ^4^ College of Pharmacy, Zunyi Medical University, Zunyi, Guizhou, China; ^5^ College of Pharmacy, Southern Medical University, Guangzhou, Guangdong, China; ^6^ University of Chinese Academy of Sciences, Beijing, China; ^7^ College of Food Science, Shenyang Agricultural University, Shenyang, Liaoning, China; ^8^ Department of Cardiology, Zhongshan Hospital Affiliated with Guangzhou University of Chinese Medicine (Zhongshan Hospital of Traditional Chinese Medicine), Zhongshan, Guangdong, China; ^9^ Guangzhou University of Chinese Medicine, Guangzhou, China; ^10^ College of Life Sciences, China Jiliang University, Hangzhou, Zhejiang, China

**Keywords:** myocardial ischemia–reperfusion injury, pathogenesis, traditional Chinese medicine, cardioprotective effects, molecular mechanisms

## Abstract

Ischemic heart disease (IHD) is a high-risk disease in the middle-aged and elderly population. The ischemic heart may be further damaged after reperfusion therapy with percutaneous coronary intervention (PCI) and other methods, namely, myocardial ischemia–reperfusion injury (MIRI), which further affects revascularization and hinders patient rehabilitation. Therefore, the investigation of new therapies against MIRI has drawn great global attention. Within the long history of the prevention and treatment of MIRI, traditional Chinese medicine (TCM) has increasingly been recognized by the scientific community for its multi-component and multi-target effects. These multi-target effects provide a conspicuous advantage to the anti-MIRI of TCM to overcome the shortcomings of single-component drugs, thereby pointing toward a novel avenue for the treatment of MIRI. However, very few reviews have summarized the currently available anti-MIRI of TCM. Therefore, a systematic data mining of TCM for protecting against MIRI will certainly accelerate the processes of drug discovery and help to identify safe candidates with synergistic formulations. The present review aims to describe TCM-based research in MIRI treatment through electronic retrieval of articles, patents, and ethnopharmacology documents. This review reported the progress of research on the active ingredients, efficacy, and underlying mechanism of anti-MIRI in TCM and TCM formulas, provided scientific support to the clinical use of TCM in the treatment of MIRI, and revealed the corresponding clinical significance and development prospects of TCM in treating MIRI.

## 1 Introduction

Cardiovascular disease (CVD) is one of the leading causes of death worldwide. As the most common CVD, ischemic heart disease (IHD) has the highest morbidity and mortality rate (Solola et al., 2022). Therefore, effective treatment for IHD is essential to reduce CVD-caused death, while the current existing therapies, such as coronary artery bypass graft, thrombolysis, and percutaneous coronary intervention (PCI), successfully reduce IHD mortality to some extent (Bittl et al., 2022). Post-therapy myocardial ischemia–reperfusion injury (MIRI) potentially causes severe myocardial damage and dysfunction, leading to post-IHD disability (Zhao H. et al., 2021). As a result, MIRI has been considered a major treatment burden for patients with IHD, and reducing myocardial ischemia–reperfusion injury becomes a clinical challenge.

The mechanism of MIRI is linked to multiple factors, and so far, research has mainly focused on oxidative stress (Xiang et al., 2021), inflammation (Yao et al., 2022), calcium overload (Wang F. Y. et al., 2020), energy metabolism disorders (Tian et al., 2019), pyroptosis (Shi H. et al., 2021), and ferroptosis (Fang et al., 2019). Targeting the factors mentioned, a range of drugs act by scavenging free radicals, ischemic preconditioning; inhibiting the exchange between Na^+^/H^+^ and Na^+^/Ca^2+^; and using adenosine receptor agonists, magnesium, statins, and angiotensin receptor antagonists to attenuate MIRI. However, acting on a single aspect is considered the main cause of unsatisfactory therapeutic outcomes of the aforementioned medicines. In contrast, traditional Chinese medicine (TCM) potentially offers a novel avenue for MIRI treatments based on their advantages of being multi-component and multi-target.

With a long history of medical use, mainly by the Chinese population, TCM has its own unique scientific theory and practical application. Various forms of herbal medicine provide enormous resources for developing a new drug against MIRI. TCM elicits potent cardiovascular protective properties, such as anti-inflammation, antioxidant effect, and immune regulation. In recent years, several studies have reported the prevention and treatment of CVD using TCM, which has achieved considerable success in research and clinical use (Leung and Xu, 2020; Zhang et al., 2020). The well-known concept of “Huo Xue Hua Yu” in Chinese (defined as “activating blood circulation and removing blood stasis” in English) of TCM formulas has been successfully applied to treat diseases by antiplatelet or anti-thrombotic activities (Zhao et al., 2020). Representative herbs include *Salvia miltiorrhiza* Bge. (or “Danshen”) (Li C. L. et al., 2020) and *Panax notoginseng* (or “Sanqi”) (Wang D. et al., 2021), which have been widely used in the treatment of CVD for a long time, not only in China but also in other Asian countries and regions. As the pharmacological properties of TCM have been increasingly studied and better understood in medical research using cutting-edge technology, greater interest has been drawn to the mechanism by which TCM contributes to MIRI treatment and the development of novel TCM-based drugs. This review aims to summarize the application of TCM to the treatment of MIRI. Four different research areas will be addressed: 1) pathological mechanisms of myocardial ischemia under the background of modern medicine, 2) mechanisms of MIRI in Chinese medicine theories, 3) single Chinese herbs and their active ingredients, and 4) TCM formulas for MIRI treatment. This review provides scientific support for the utilization and future development of TCM resources and provides a broad prospect for TCM in the treatment of myocardial ischemia through the comprehensive analysis of various kinds of TCM against MIRI.

## 2 Research progress on the mechanisms of MIRI

### 2.1 Pathological mechanism of MIRI in modern medicine

Blood reperfusion is an essential process during the treatment of myocardial ischemia, but it may also further aggravate damage or destroy cardiac function, inducing severe arrhythmias or even leading to sudden death (Wang J. et al., 2020). The severity of the reperfusion injury is dependent on many factors, such as the length of ischemia time, the temperature and pressure of the perfusion fluid, and the state of ischemic tissues and organs. From the perspective of cardiac function, the resting tension gradually increases with the prolongation of ischemia time, and the developed tension gradually decreases, while the resting tension increases and the developmental tension becomes lower during reperfusion, indicating that the systolic force of the heart decreases diastolic and systolic dysfunction of the heart (He et al., 2022).

Reperfusion therapy improves myocardial blood supply, but it is accompanied by a complex series of pathophysiological responses, such as oxidative stress, inflammatory response, calcium overload, and mitochondrial dysfunction, of which the key driving factor that causes reperfusion injury is oxidative stress (Zheng et al., 2021).

#### 2.1.1 Oxidative stress

Oxidative stress is a consequence of imbalanced redox systems in the body, which lead to the accumulation of oxygen radicals and a decrease in the activity of antioxidant enzymes, triggering lipid peroxidation and causing cell damage. During myocardial ischemia, there is an insufficient supply of oxygen and ATP cannot be supplied adequately, impairing the ability of the myocardium to clear free radicals. When blood supply is restored by reperfusion, the large accumulation of oxygen radicals leads to lipid peroxidation, producing malondialdehyde (MDA). MDA triggers cytotoxicity and affects the mitochondrial respiratory chain complex and key enzyme activities within the mitochondria (Xiang et al., 2021). In addition, oxygen radicals can destabilize body proteins through oxidation, thereby altering the surface structure of proteases. Oxygen radicals can also induce apoptosis, which is caused by the breakage of DNA/RNA in cells, making nucleic acids non-functional (Granger and Kvietys, 2015).

#### 2.1.2 Calcium overload

Calcium ions (Ca^2+^) are intracellular secondary messengers involved in maintaining cellular physiological functions. Ca^2+^ overload is both a result of myocardial damage and a cause of further damage. Upon myocardial ischemia, the myocardial membrane structure is damaged, and the membrane becomes more permeable to Ca^2+^. Increasing entry of Ca^2+^ from the extracellular space, following a concentration gradient, leads to myocardial Ca^2+^ overload. Cells switch from aerobic to anaerobic respiration during myocardial ischemia, which increases lactate production and decreases intracellular pH. Activation of the Na^+^–H^+^ exchanger leads to a marked increase in intracellular Na^+^ concentration. Reperfusion rapidly restores extracellular pH, resulting in a significant difference in intra- and extracellular pH, causing a large inward flow of Na^+^ through the Na^+^–H^+^ exchanger and an excess of intracellular Na^+^, triggering a reversal of the 2Na^+^–Ca^2+^ exchanger, and resulting in intracellular Ca^2+^ overload (Wang L. et al., 2020; Wang C. et al., 2022).

#### 2.1.3 Mitochondrial dysfunction

Mitochondria act as the body’s energy engine, and most of the body’s energy is produced by mitochondrial oxidative phosphorylation. Mitochondria have been shown to play an important role in the progression of MIRI. Ischemia leads to the interruption of oxidative phosphorylation, which causes a rapid decrease of adenosine triphosphate (ATP) and creatine phosphate (CP) in the myocardium, resulting in an excess of non-phosphorylated purines in cardiomyocytes. The non-phosphorylated purines enter blood vessels and subsequently block the generation of ATP (Bai et al., 2021). After reperfusion, nucleosides are significantly reduced by the reduction of the raw material for the synthesis of high-energy phosphoric acid compounds in the myocardium. This process results in impaired mitochondrial energy metabolism, which affects the recovery of cardiac function. Acidic conditions during ischemia prevent the mitochondrial permeability transition pore (mPTP) from opening (Marzilli et al., 2020). During reperfusion, the electron transport chain is reactivated to produce reactive oxygen species (ROS) (Martins-Marques et al., 2021). ROS act as a neutrophil chemoattractant by inducing mPTP opening and sarcoplasmic reticulum (SR) dysfunction, thereby mediating myocardial reperfusion injury (Andrienko et al., 2017). After reperfusion, the restored physiological pH would relieve the inhibition of mPTP opening and cardiomyocyte contracture (Lin K. et al., 2015). The recovery of mitochondrial membrane potential promotes calcium entry into the mitochondria and induces mPTP opening. Several hours after the onset of myocardial reperfusion, neutrophils accumulate in the infarcted myocardium in response to the release of chemical attractant ROS, cytokines, and activated complements.

#### 2.1.4 Inflammatory response

The inflammatory response is also an important part of MIRI. The membrane structures of myocardial endothelial tissue are damaged during MIRI and attract large numbers of neutrophils into the tissue or adhere to the myocardial vascular endothelium. The accumulation of neutrophils can lead to the release of IL-6, TNF-α, and IL-1β, causing an inflammatory response and inducing microcirculatory disturbances, which can lead to myocardial injury (Sun X. et al., 2021).

#### 2.1.5 Cell death

**FIGURE 1:**
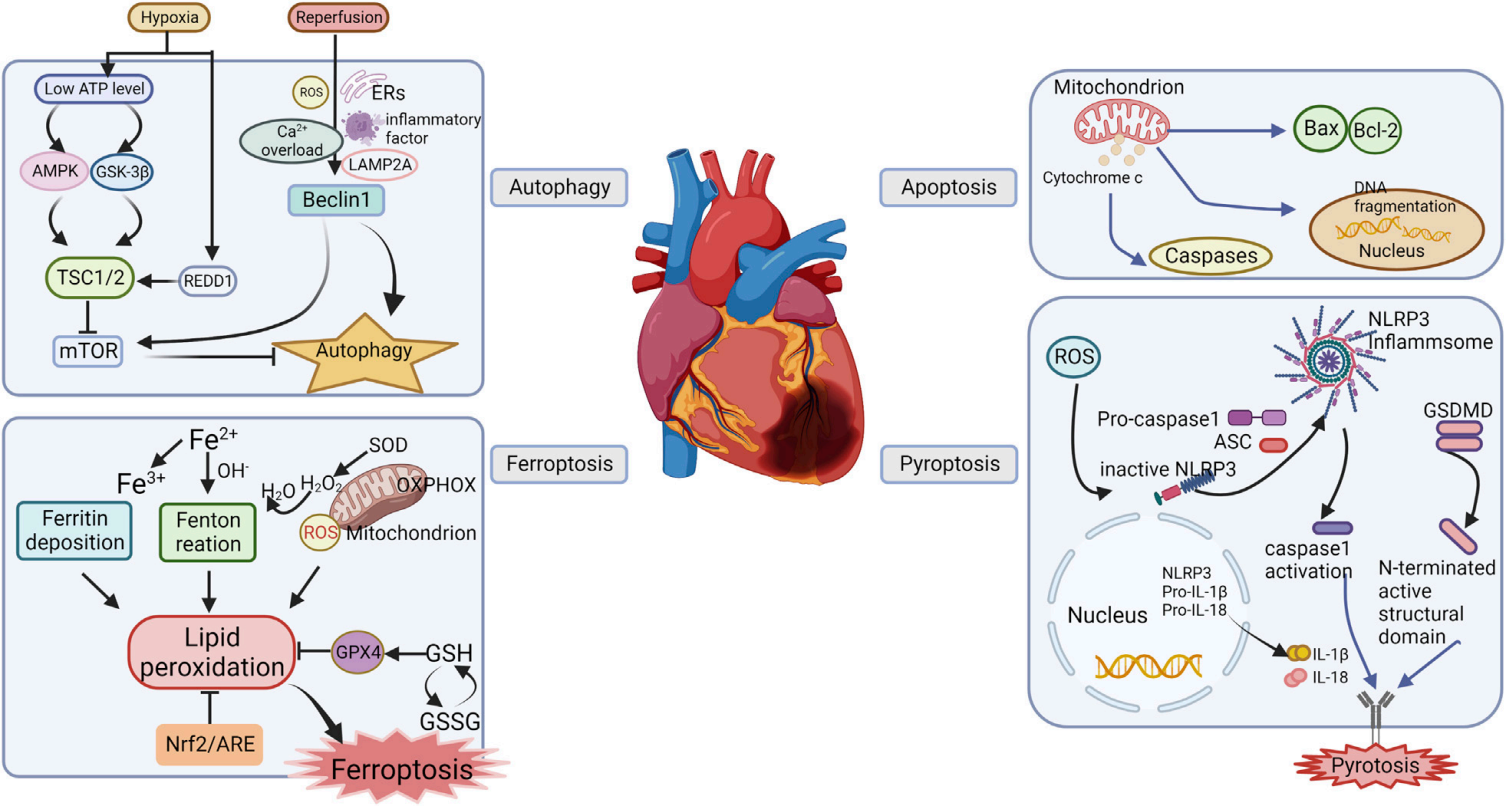
Mechanisms of programmed cell death of MIRI.

Cell death is also a consequence of ischemia–reperfusion injury during MIRI. Cell death pathways include autophagy, ferroptosis, pyroptosis, and apoptosis, which are regulated by a variety of signaling pathways that affect the state of the cell (Figure 1).

##### 2.1.5.1 Autophagy in MIRI

Autophagy is a process of phagocytosis and degradation that maintains cell homeostasis. It is a key regulator of ischemia/reperfusion (I/R) injury and is believed to play important roles in the heart during I/R, which can be activated by several stimuli including nutrient starvation, glucose deprivation, caloric restriction, oxidative stress, and brief episodes of ischemia and reperfusion to eliminate damaged mitochondria. Autophagy prevents damaged mitochondria from releasing harmful ROS, decreases apoptosis, and helps maintain mitochondrial homeostasis and cell life activities through the related pathways, mediated by the mammalian rapamycin target protein (mTOR) (Shi et al., 2019). Under the condition of myocardial ischemia, the AMPK–mTOR pathway is activated and initiates autophagy, which would swallow damaged mitochondria and reduce cardiomyocyte damage due to mitochondrial dysfunction and ATP depletion (Gatica et al., 2015). As myocardial energy is depleted, the AMP/ATP ratio increases, which causes GSK-3 activation and downregulation of the expression of the tuberous sclerosis complex 1/2 TSC1/2, thereby inhibiting mTOR signaling (Zhai and Sadoshima, 2011). However, studies have also reported that prolonged ischemia could trigger excessive autophagy and lead to cardiomyocyte death, which further deteriorates cardiac function (Rabinovich-Nikitin et al., 2021).

During myocardial reperfusion, a burst of oxygen-derived free radicals could lead to excessive autophagy that contributes to autophagic cell death and aggravates myocardial injury (Yang H. et al., 2021). Recombinant lysosomal-associated membrane protein 2 (LAMP2) is a critical factor in autophagosome–lysosome fusion, whose decline can lead to an increase in autophagosomes. Thus, upregulating the expression of LAMP2 during reperfusion suppresses autophagy and attenuating myocardial infarction (Ghosh et al., 2022). Furthermore, the effect of autophagy on the protection or aggravation of injury by reperfusion may be related to the expression of Beclin 1, and mTOR also mediates interaction with Beclin 1 through caspase-3, which plays a key role in autophagy (Dong et al., 2019). The Ca^2+^ overload during reperfusion promotes CaMKII, which phosphorylates Beclin 1 at Ser90, subsequently promoting the ubiquitination of Beclin 1 at the K63 site to cause excessive autophagy and cell death (Chen et al., 2021).

##### 2.1.5.2 Pyroptosis in MIRI

Pyroptosis, a pro-inflammatory cell death process, is mediated by gasdermin D (GSDMD) or gasdermin E (GSDME). ROS could activate the NOD-like receptor protein 3 (NLRP3) inflammasome and then induce the activation of caspase enzymes, which is an important process of MIRI-mediated pyroptosis (Bortolotti et al., 2018). On the one hand, GSDMD is cleaved by activated caspase-1 to form a polypeptide containing the nitrogen-terminated active structural domain of GSDMD (Wang et al., 2020d). On the other hand, apoptosis-associated speck-like protein containing a caspase recruitment domain (ASC) induces pro-caspase-1 inflammasome formation and results in caspase-1 activation. Activated caspase-1 cleaves the precursors of IL-1β and IL-18 to their active forms (Wang et al., 2020e). These processes promote the accumulation of inflammatory cells, which results in pyroptosis.

##### 2.1.5.3 Ferroptosis in MIRI

Ferroptosis is an iron-dependent, non-apoptotic form of cell death that is characterized by iron overload and lipid peroxidation. Ferroptosis is critical for the pathogenesis of MIRI. In the transplant model, ferrostatin-1 (FER-1) has been found to protect the heart from MIRI by reducing the levels of hydrogen peroxide-arachidonic acid-phosphatidylethanolamine, which is an intermediate product that mediates iron death; in this way, FER-1 reduces the death of cardiomyocytes and myocardial fibroblasts. At the same time, iron death specifically promotes the adhesion recruitment of neutrophils to coronary endothelial cells through the TLR4/TRIF/IFN-1 signaling pathway, which aggravates heart damage (Li W. et al., 2019). Meanwhile, in rat models of diabetic MIRI, the DNA methyltransferase-1 (DNMT-1) inhibitor 5-aza-2′-deoxycytidine (5-aza-CdR) has been reported to reduce nuclear receptor coactivator 4 (NCOA4)-mediated ferritin phagocytosis and myocardial damage. Inhibiting DNMT-1 reduces iron death during DM, and NCOA4-mediated phagocytosis of ferritin is potentially involved in this process (Li Q. et al., 2020). Another study showed that the expression of ubiquitin-specific protease 7 (USP7) in rat myocardial tissue with MIRI was significantly upregulated. The expressions of P53 (the human tumor suppressor gene) and transferrin receptor 1 (TfR1) also increased along with the exacerbation of iron death in myocardial tissue (Tang et al., 2021). Further study based on an artificial model of glutathione peroxidase 4 (GPX4) deletion in the proximal tubules indicated that knockout of GPX4 caused cell death in a pathologically relevant form of ferroptosis and spontaneous tubular necrosis, and the inhibition of ferroptosis by liproxstatin-1 was able to mitigate I/R-induced tissue damage (Park et al., 2019).

### 2.2 Mechanism of MIRI under the background of Chinese medicine theories

“Myocardial ischemia-reperfusion injury” is not defined in the theory of Chinese medicine, but based on its lesion location (heart collateral and heart pulse) and clinical manifestations (chest tightness, chest pain, shortness of breath, and fatigue), MIRI is classified into “chest paralysis,” “heartache,” “palpitation,” and other categories (Xie et al., 2015). Chinese medicine practitioners believe that the core of MIRI pathogenesis is heart vessel blockage caused by various etiologies. Although MIRI originates from heart disease, it also affects the lungs, liver, kidney, spleen, and other organs. The nature of MIRI in Chinese medicine mainly lies in two aspects of root vacuity and tip repletion, which interact with each other and jointly affect the pathological process of MIRI. Among them, root vacuity refers to a deficiency of qi and insufficient blood circulation and Yin and Yang, while tip repletion refers to phlegm turbidity, cold coagulation, qi stagnation, blood stasis, and other phenomena (Li et al., 2021). The TCM pathogenesis of MIRI includes qi deficiency, blood stasis, and phlegm turbidity. Qi deficiency leads to the formation of blood stasis and turbid phlegm, which further aggravates the degree of qi deficiency, forming a vicious circle. The pathogenesis of the disease and the severity of the disease are the trends of MIRI. Based on relative documents and clinical symptoms of patients with MIRI, Chinese medicine has divided MIRI into two main types: turbid phlegm obstruction syndrome and qi and blood stasis obstruction. From a patient’s constitution of different types of symptoms, a multifaceted diagnosis is made to find the optimal treatment program, which has the features and advantages of Chinese medicine treatment of MIRI.

#### 2.2.1 Turbid phlegm obstruction syndrome

Some scholars believe that the etiology and pathogenesis of acute myocardial ischemia are caused by the organ’s dysfunction and qi–blood–body fluid generation, resulting in the turbidity of phlegm. Phlegm turbidity directly attached to the vein wall, resulting in abnormal qi–blood circulation, blood stasis, phlegm–blood stasis, and heart vessel blockage stasis, hinders the operation of meridians and collaterals and thus causes microcirculation disorders (Ma et al., 2013). After subsequent reperfusion, distal blood flow is greatly reduced, causing reperfusion injury. Microthrombus and small atherosclerotic plaques would block distal vessels with blood flow after reperfusion, causing insufficient blood flow in distal vessels and aggravating injuries (Bai and Ren, 2014; Liu et al., 2014). Body fluid is homologous to blood in the body. Phlegm and stasis are the pathological products of blood fluid. Qi–blood stasis and stagnant movement of qi and blood will affect water metabolism. In the initial stage of reperfusion, rapid blood flow is likely to induce water retention, forming “edema” and “phlegm drinking” in cells, which leads to slow blood flow in vessels (Zhu et al., 2015).

#### 2.2.2 Qi and blood stasis syndrome

The blood and oxygen supply are cut off during myocardial ischemia, which leads to myocardial dystrophy and causes varying degrees of insufficiency of the heart qi. The relationship between qi and blood is one of mutual sustenance, interdependence, and mutual use, and blood has the role of moistening and carrying qi.

If there is insufficiency of the heart qi for a long time, blood vessels may show restenosis symptoms due to poor blood flow and venous stasis. Suddenly, large amounts of blood await delivery to distal tissues during reperfusion, instead increasing the burden on the heart, depleting the qi of the heart and leading to reperfusion injury (Wang and Yan, 2016). Qi deficiency inevitably leads to weakened qi transformation function, an inability to complete the mutual transformation of qi, blood, and fluid, and an inability to transform blood into nutrients for tissue use, resulting in a lack of nutrients for cardiac muscle cells and increased cardiac function damage, causing myocardial apoptosis, and thus symptoms of cardiac stasis.

The myocardial tissue will lead to myocardial qi deficiency due to ischemia and hypoxia; however, qi deficiency of the heart results in slow blood flow. Therefore, after reperfusion, the heart is unable to deliver blood quickly to the distal tissues for use, resulting in blood stasis in the distal vessels. In this case, the myocardium becomes deficient in qi and blood, which is called “pulse knotting and palpitations” in Chinese medicine, just like reperfusion arrhythmia in Western medicine (Li and Zhu, 2012).

## 3 Herb medicines and their active chemical ingredients in TCM formulas for MIRI treatment

**FIGURE 2:**
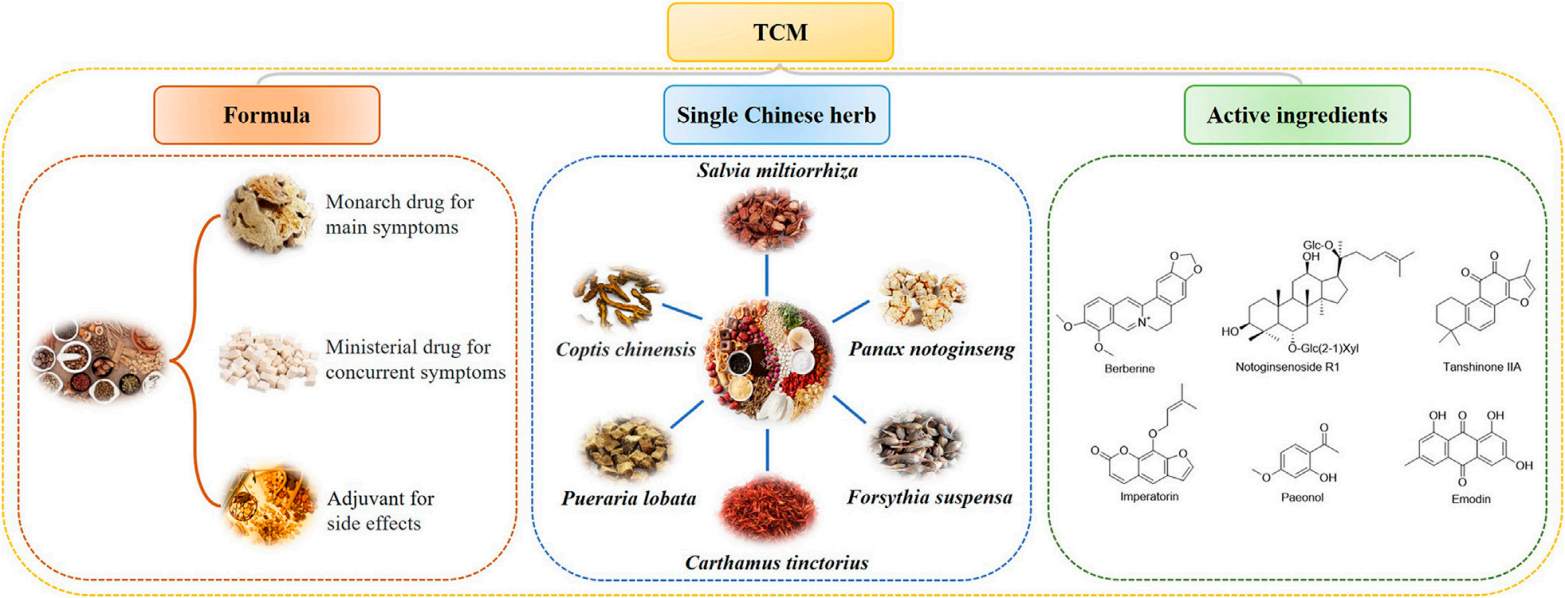
TCM formulas, single Chinese herbs, and active compounds that protect the heart from MIRI.

**FIGURE 3:**
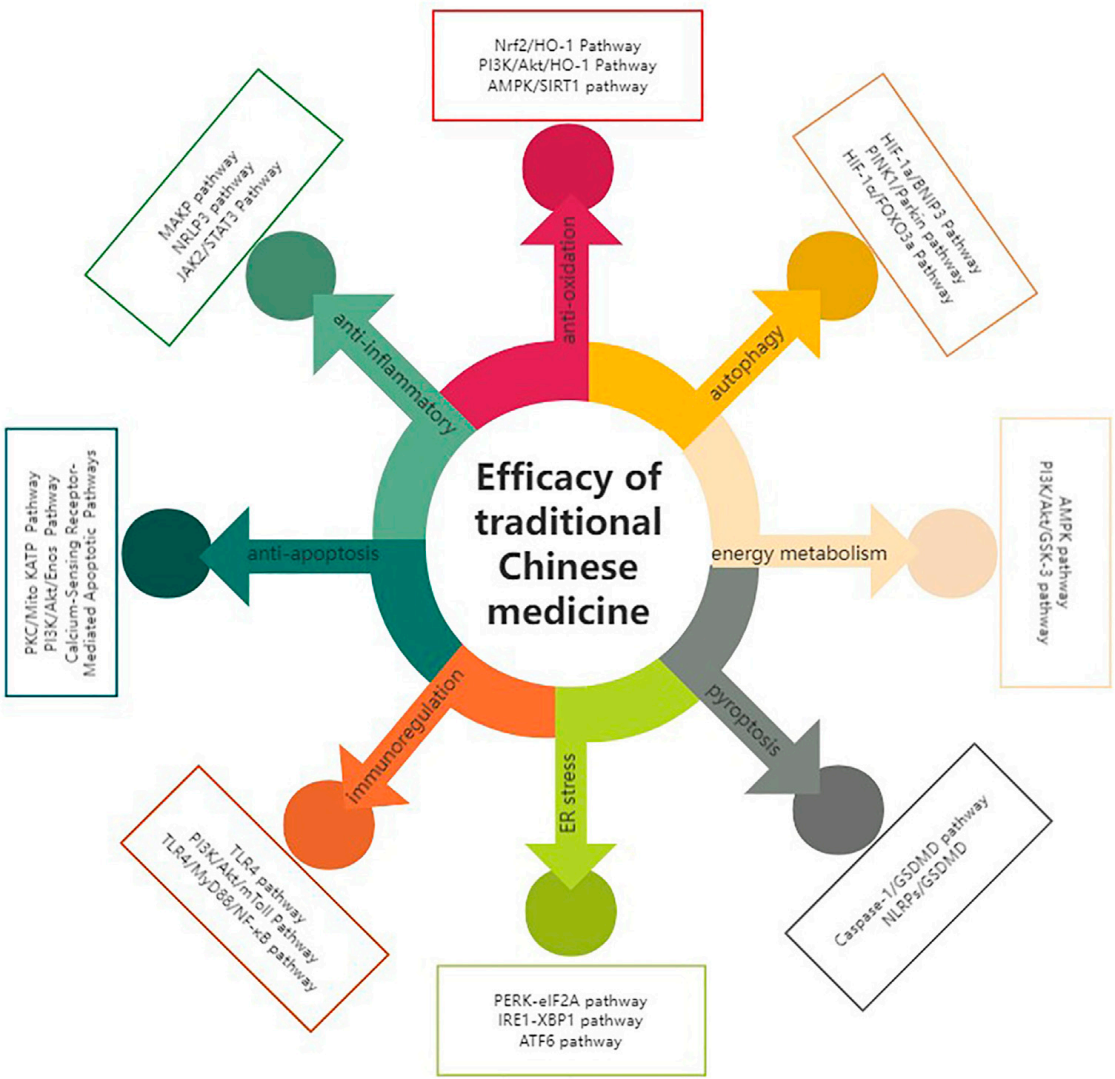
TCM anti-MIRI effects and their respective pathways.

TCM plays an important and indispensable role in preventing and treating MIRI. Some Chinese patent drugs have shown significant myocardial protection in MIRI. The chemical components of TCM are structurally diverse and have multiple pharmacological effects. Single Chinese herbs and their chemical constituents, TCM formulas, and TCM-based prescriptions may have therapeutic efficacy against MIRI (Tables 1,2). Classifying and analyzing the active ingredients of TCM reported in previous literature can provide a reference for determining the research trends in this field in the future (Figures 2,3).

**TABLE 1:** Medicinal materials and their chemical constituents for the therapy of MIRI.

TCM	Chinese medicine theories	Active ingredients	Anti-MIRI experiments	Animal or cell	Dose range	Time of dosing	Efficacy	Mechanism of actions	References
*Salvia miltiorrhiza* (Danshen)	Activating blood and removing stasis	Tanshinone IIA	I/R model (30 min/2 h)	SD rats	10 mg/kg	9 weeks	Anti-fibrosis	Decrease in mRNA expressions of collagen I, collagen III, TIMP-1, and galectin-3. Increase in mRNA and protein expression of MMP-2	Zhang et al. (2020)
			I/R model (30 min/2 h)	SD rats	1.5 mg/kg	3 h	Anti-inflammatory	Increase in protein expressions of P-JAK2, P-STAT3, cleaved caspase-3, and the Bcl-2/Bax ratio	Wang et al. (2021a)
		Sodium tanshinone IIA sulfonate (STS)	I/R model (45 min/2 h)	SD rats	0.5 mg/kg, 1 mg/kg, and 2 mg/kg	8 weeks	Anti-apoptosis	Increase in the expressions of Bcl-2, P-p65, IL-8, IL-10^+^, and TNF-α. Decrease in the expressions of Bax and iNOS^+^, and increase in improved Th1/Th2 balance	Li et al. (2019a)
			I/R model (45 min/2 h)	SD rats	8 mg/kg and 16 mg/kg	2.5 h	Anti-inflammatory	Decrease in IL-23 and IL-17, IL-1β, and HMGB1 release, and inhibition of MPO activity	Ding (2020b)
		Cryptotanshinone (CTS)	I/R model (45 min/2 h)	SD rats	40 mg/kg	3 days	Anti-inflammatory and inhibiting ER stress	Increase in the expressions of p-JAK1 and p-STAT3. Decrease in the expressions of CHOP, GRP78, and caspase-12	Wang et al. (2013)
		Dihydrotanshinone I (DT)	OGD model (8 h/2 h)	H9c2 cells	1 μM	1 h, 2 h, 4 h, 8 h	Anti-oxidation	Decrease in the expressions of NADH and HIF-1α, and increase in the expression of Nrf2	Jiang et al. (2019)
		Protocatechuic aldehyde (PCA)	OGD/R model (4 h/24 h)	H9c2 cells	1.25 μM, 2.5 μM, and 5.0 μM	28 h	Inhibiting ER stress	Decrease in the expressions of PERK, IRE1α, and ATF6α	Wan et al. (2021)
		Salvianolate	I/R model (30 min/2 h)	SD rats	10 mg/kg, 20 mg/kg, and 40 mg/kg	7 days	Anti-oxidation	Increase in the expression of Bcl-2, and decrease in the expressions of Bax and cleaved caspase-3	Li (2019)
		Salvia magnesium lithospermate B	I/R model (1 h/3 h)	SD rats	10 mg/kg and 30 mg/kg	3.5 h	Anti-apoptosis	Decrease in the expressions of RIPK1 and RIPK3	Wang et al. (2017)
		Danshensu	OGD/R model (6 h/18 h)	H9c2 cells	10 μM	1 h	Anti-apoptosis	Increase in Akt and ERK1/2 mRNA and protein expressions, and decrease in miR-199a-5p mRNA expression	Shi et al. (2021a)
			I/R model (1 h/3 h)	SD rats	10 μM	1 h	Anti-oxidation	Decrease in ROS level, increase in Sirt1 and Bcl-2 mRNA expressions, and decrease in FoxO1 and Rab7 mRNA expressions	Sun et al. (2020)
		Salvianolic acid A	I/R model (45 min/3 h and 45 min/24 h)	SD rats	10 mg/kg	3 h and 24 h	Anti-inflammatory	Reduced serum levels of p-selectin, TNF-α, IL-1β, and NO.	Yuan et al. (2017)
		Salvianolic acid B	I/R model (3 h/24 h)	SD rats	15 mg/kg and 60 mg/kg	4 days	Anti-apoptosis	Increase in the expressions of Bcl-2 and P-Akt and decrease in the expressions of Bax, HMGB1, and TLR4	Liu et al. (2020c)
*Panax notoginseng* (Sanqi)	Blood-activating and stasis-eliminating compound, dilated blood vessels, and improved microcirculation	*Panax notoginseng* saponins (PNS)	I/R model (30 min/7 days)	SD rats	30 mg/kg and 60 mg/kg	7 days	Anti-apoptosis	Increase in the expression of LC3 and the ratio of LC3II/LC3I, and increase in the expressions of HIF-1a, BNIP3, Atg5, and Beclin-1	Liu et al. (2019)
			H/R model (18 h/6 h)	H9c2 cells	400 μg/ml	24 h	Anti-apoptosis	Increase in the expressions of HIF-1a, BNIP3, FOXO3a, and Akt protein; increase in protein LC3 and the ratio of LC3II/LC3I; and decrease in the expression of Bim	Liu et al. (2021c)
			H/R model (4 h/2 h)	H9c2 cells	200 μg/ml and 500 μg/ml	12 h	Anti-oxidation	Increase in the expression of miR-30c-5p	Wang et al. (2020a)
		Notoginsenoside R1 (NGR1)	H/R model (6 h/12 h)	H9c2 cells	1 μM	24 h	Anti-apoptosis	Decrease in the protein expressions of GRP78, P-PERK, ATF6, IRE, CHOP, caspase-12, and P-JNK.	Yu et al. (2016)
			I/R model (30 min/7 days)	SD rats	20 mg/kg and 40 mg/kg	5 days	Anti-inflammatory and anti-apoptosis	Decrease in the level of IL-1β, IL-8, and TNF-α in serum; decrease in the expressions of caspase 3 and Bax; and increase in the expressions of Bcl-2, PI3K, and p-Akt	Zhou and Liu (2019)
			H/R model (6 h/12 h)	H9c2 cells	20 μM	24 h	Anti-oxidation	Increase in the expression of miR-132, and decrease in the expression of HBEGF	Jin et al. (2021a)
		Ginsenoside Rg1 (Rg1)	I/R model (30 min/90 min)	SD rats	5 mg/kg	2.5 h	Anti-apoptosis and modulating energy metabolism	Increase in the ratio of Bax/Bcl-2 and the expressions of cleaved caspase-3, ECH1, and ENOβ, and decrease in the expressions of HIF1, ENOα, ALDOA, and phosphorylation level of MYPT-1 and MLC	Li et al. (2018)
*Astragalus membranaceus* (Huangqi)	Replenishing qi and nourishing blood, tonifying qi, and strengthening exterior	Astragaloside-IV (AS-IV)	H/R model (2 h/24 h)	Neonatal cardiac myocyte	60 μM	24 h	Anti-apoptosis	Decrease in the expressions of Bax, cleaved caspase-3, and CaSR, and increase in the ratio of Bcl-2 and p-ERK/ERK.	Yin et al. (2019)
			H/R model (5 h/1 h)	AC16 cells	20 μM, 40 μM, and 80 μM	96 h	Anti-apoptosis	Increase in the expressions of miR-101a and Bcl-2, and decrease in the expressions of TGFBR1, TLR2, Bax, cleaved caspase-3, p-ERK, and p-p38	Wu et al. (2020)
			H/R model (12 h/8 h)	H9c2 cells	100 μM	20 h	Anti-apoptosis and anti-oxidation	Increase in the expressions of PI3K, p-Akt, HO-1, and Nrf2, and decrease in the expression of Bach1	Yang et al. (2019)
			H/R model (12 h/8 h)	H9c2 cells	50 μM	20 min	Inhibit ER stress	Decrease in the expressions of GRP 78, GRP 94, and IRE1	Li et al. (2017a)
		Astragalus polysaccharides (ASP)	Isoprenaline (ISO) for 48 h	H9c2 cells	5 μg/L, 10 μg/L, and 20 μg/L	48 h	Anti-apoptosis	Decrease in the protein levels of caspase-3 and Bax, and increase in the protein levels of bcl-2	Liu et al. (2018b)
			H/R model (2 h/4 h)	MMECs	25 μg/L, 50 μg/L, and 100 mg/L	2 h	Anti-apoptosis	Increase in the expressions of p-PI3K, p-Akt, eNOS, and p-eNOS.	Zhou et al. (2018)
*Scutellaria baicalensis* (Huangqin)	Heat-clearing and damp-drying	Wogonoside (WG)	I/R model (30 min/24 h)	SD rats	20 mg/kg and 40 mg/kg	24 h	Anti-oxidation	Increase in the expressions of Nrf2 and HO-1 mRNA and protein	Li et al. (2021)
			H/R model (4 h/6 h)	H9c2 cells	12.5 μM, 25 μM, and 50 μM	24 h	Anti-inflammatory and anti-apoptosis	Increase in the expression of Bcl-2, and decrease in the expression levels of Bax, caspase-3, caspase-9, IL-6, IL-1β, iNOS, p38, and ERK1/2	Song et al. (2019)
		Baicalin	H/R model (6 h/12 h)	H9c2 cells	10 μM	1.5 h	Anti-oxidation and anti-apoptosis	Decrease in the expression of caspase-3, and increase in the expression levels of ALDH2 mRNA and protein and the activity of ALDH2	Jiang et al. (2018)
			I/R model (30 min/3 h)	C57 mice	25 mg/kg	3.5 h	Anti-oxidation	Increase in the expressions of MARCH5, LC3-II, and KLF4, and decrease in the expression of Drp1	Li et al. (2020a)
*Rheum palmatum* (Dahuang)	Breaking stagnation and blood stasis	Emodin (Emo)	I/R model (30 min/48 h)	SD rats	20 mg/kg, 40 mg/kg, and 60 mg/kg	10 days	Anti-oxidation	Increase in the expressions of Nrf2 and HO-1	Cui et al. (2020)
			H/R model (1 h/2 h)	Rat primary cardiomyocytes	2.5 μM, 5 μM, and 10 μM	1 h	Anti-inflammatory and anti-pyroptosis	Decrease in the expressions of IL-1β, TLR4, MyD88, phospho-IκBα, phospho-NF-κB, the NLRP3 inflammasome, and GSDMD-N	Ye et al. (2019)
		Rhein	H/R model (6 h/2 h)	H9c2 cells	10 μM	9 h	Anti-oxidation	Increase in the phosphorylation of Akt and GSK3β, and decrease in p-P38	Liu et al. (2018a)
*Paeonia lactiflora* (Shaoyao)	Activating blood and dieresis	Paeonol	I/R model (40 min/120 min)	SD rats	12 mg/kg	24 h	Anti-apoptosis	Increase in the expression of SIRT1	Liu et al. (2020a)
			I/R model (1 h/3 h)	SD rats	0.1 mg/kg and 1 mg/kg	4 h	Anti-apoptosis and regulating autophagy	Decrease in the cleaved forms of caspase-8, caspase-9, caspase-3 and PARP, Beclin-1, p62, LC3-I, and LC3-II protein expressions, and increase in the Bcl-2/Bax and Bcl-2/Beclin-1 ratios	Tsai et al. (2021)
*Hypericum perforatum* (Lianqiao)	Clearing away heat and reducing swelling	Hyperoside (Hyp)	H/R model (8 h/2 h)	Neonatal rat cardiomyocytes	25 μM	12 h	Anti-apoptosis	Decrease in Bnip3, Bax, cleaved caspase 3, TLR4, and CREB protein expressions, and increase in expression of Bcl-2	Xiao et al. (2017)
			H/R model (8 h/2 h)	H9c2 cells	50 μM	12 h	Anti-apoptosis	Increase in the expressions of PKCε, Nrf2, and Kir6.2, and decrease in the expression of caspase-3	Wang et al. (2020b)
*Erigeron breviscapus* (Dengzhanxixin)	Dispelling wind and eliminating dampness, promoting blood circulation to remove blood stasis, dredging channels, and activating blood circulation to dissipate stasis	Breviscapine	H/R model (8 h/16 h)	MEC cells	50 μM	24 h	Anti-apoptosis and regulating autophagy	Decrease in the expressions of ICAM-1mRNA and VCAM-1 mRNA, increase in LC3 mRNA and protein, decrease in TLR4 and caspase-3, and increase in CREB	Wang et al. (2021b)
			I/R model (30 min/2 h)	SD rats	100 mg/kg and 200 mg/kg	14 days	Anti-apoptosis and anti-inflammatory	Decrease in the expressions of IL-1β, IL-6, TNF-α, LC3-II/LC3-I, and Beclin1, and increase in the expressions of mTOR, p-PI3K, and p-Akt	Yang et al. (2021a)
*Phellodendron chinens* (Huanglian)	Clearing heat and damp-drying	Berberine	I/R model (30 min/14 h)	SD rats	75 mg/kg and 150 mg/kg	14 days	Regulating autophagy	Increase in the expressions of PINK1, Parkin, LC3B, p62, and USP30	Sun et al. (2021a)
*Crataegus pinnatifida* (Shanzha)	Activating blood circulation to dissipate stasis	Vitexin	I/R model (30 min/30 min)	SD rats	1 μM, 3 μM, 10 μM, and 30 μM	1.5 h	Anti-apoptosis	Increase in the expression of Bcl-2, and decrease in the expressions of Bax, cleaved caspase-3/9, cytochrome c, Bax, SDHB, COX IV, MFN2, and Mito-Drp1	Xue (2020)
			H/R model (5 h/1 h)	H9c2 cells		2 h		Decrease in the expressions of cleaved caspase-3/9, ROS, NOX4, Cyt-c, SDHB, COX IV, MFN2, and Mito-Drp1	
			I/R model (30 min/30 min)	SD rats	10 μM	20 min	Anti-apoptosis and regulation of mitochondrial dynamics	Increase in the expressions of cleaved caspase-3, cleaved caspase-9, Epac1, Rap1, and Bcl-2, and decrease in the expression of Bax	Yang et al. (2021b)
			H/R model (5 h/2 h)	H9c2 cells		5 h		Decrease in the expression of NOX4, and increase in the expressions of MFN2 and Drp1	
*Rhodiola rosea* (Hongjingtian)	Enriching qi, activating blood, and eliminating stasis to subdue swelling	Salidroside	I/R model (30 min/2 h)	SD rats	40 mg/kg	2.5 h	Inhibiting ER stress, reducing mitochondrial fission, and anti-apoptosis	Increase in the expressions of AMPK, Bcl-2, Opa1, and Mfn2, and decrease in the expressions of PERK, p-eIF2α, CHOP, Bax, cleared caspase-3, p-Drp1, and Fis1	Tian et al. (2022)
			H/R model (9 h/6 h)	H9c2 cells	10 μM	15 h	Anti-apoptosis	Decrease in the expressions of P-ERK/ERK, P-eIF2a, CHOP, Bax, cleared caspase-3, and p-Drp1/t-Drp1, and increase in the expressions of P-AMPK/AMPK and Bcl-2	
			I/R model (30 min/60 min)	Wistar rats	20 mg/kg	2 h	Anti-apoptosis	Increase in the expressions of Bcl-2 and P62, and decrease in the expressions of Bax, caspase-3, caspase-9, Beclin-1, and LC3BⅡ/LC3BⅠ	Jin et al. (2021b)
			H/R model (4 h/2 h, 4 h, 6 h, 8 h, and 16 h)	H9c2 cells	10 μM	30 min	Inhibiting ER stress and anti-apoptosis	Decrease in the expressions of GRP78, CHOP, Bax, caspase-3, cleaved caspase-12, P-IRE1a/IRE1a, and P-PERK/PERK, and increase in the expression of Bcl-2	Sun et al. (2018)
*Carthamus tinctorius* (Honghua)	Activating blood to promote menstruation and eliminating stasis to stop pain	Hydroxysafflor yellow A	H/R model (12 h/4 h)	H9c2 cells	5 μM	4 h	Anti-apoptosis	Increase in the expressions of p-Akt/Akt, p-GSK-3β/GSK-3β, hexokinase II, and cytochrome c	Min and Wei. (2017)
		Carthamin yellow	I/R model (30 min/6 h)	Wistar rats	10 mg/kg	3.5 h	Anti-inflammatory	Decrease in the expressions of TNF-a, IL-6, IL-1b, NLRP3, and caspase-1	Lu et al. (2019)
*Ginkgo* (Yinxing)	Promoting and dispersing lung-qi and protecting the blood vessels	Ginkgolide B	H/R model (12 h/4 h)	H9c2 cell	10 µM	24 h	Anti-apoptosis	Decrease in the expressions of Bax and cleaved caspase-3, and increase in the expressions of Bcl-2, P-Akt/Akt, and P-mTOR/mTOR	Liu et al. (2020b)
*Acorus tatarinowii* (Shichangpu)	Managing qi, activating blood, and fortifying the spleen to sweep phlegm	Beta-asarone	I/R model (45 min/24 h)	SD rats	10 mg/kg, 20 mg/kg, and 30 mg/kg	24 h	Anti-apoptosis, anti-inflammatory, and anti-pyroptosis	Decrease in the expressions of NLRP3, ASC, Cas-1, pro-Cas-1, GSDMD-F, and GSDMD-C	Xiao et al. (2020)
*Schisandra chinensis* (Wuweizi)	Tonifying qi and Yin and engendering liquid and allay thirst	Schisandrin B	I/R model (40 min/1 h)	SD rats	20 mg/kg, 40 mg/kg, and 80 mg/kg	7 days	Inhibiting ER stress and anti-apoptosis	Decrease in the expressions of CHOP, ATF6, PERK, caspase-9, caspase-3, and Bax proteins, and increase in the expression of Bcl-2	Zhang et al. (2017)

**TABLE 2:** TCM formulas for treating MIRI.

Name of formula	Theories of formula	Anti-MIRI experiments	Animal or cell	Dose range	Time of dosing	Efficacy	Mechanism of actions	Reference
Yiqi Huayu decoction (Danshen, Sanqi, Wuzhi Maotao, Xianhecao, and Hongjingtian)	Tonifying qi, nourishing Yin, and promoting blood circulation	I/R model (40 min/60 h)	SD rats	3.97 g/kg, 7.94 g/kg, and 15.9 g/kg	10 days	Anti-oxidation and anti-inflammatory	Increase in SOD activity, and decrease in TNF-α and MMP-9 level	Huang and Liao. (2012)
*Angelica sinensis* decoction (Shaoyao, Gancao, and Rougui)	Replenish blood and promote blood circulation	I/R model (30 min/2 h)	SD rats	Ferulic acid 300 mg/kg, cinnamic acid 200 mg/kg, and glycyrrhizic acid 50 mg/kg	4 days	Anti-oxidation	Increase in SOD activity, and decrease in MDA level	Liu et al. (2021a)
Buyang Huanwu decoction (Huangqi, Danghui, Cishao, Dilong, Chuanxiong, Taoren, and Honghua)	Replenishing qi, promoting blood circulation, and dredging collaterals	I/R model (30 min/120 h)	Wistar rats	15 g/kg	7 days	Anti-oxidation	Increase in SOD and NO activity, and decrease in MDA level	Song et al. (2020)
Huanglian Jiedu decoction (Huanglian, Huangqi, Huangbai, and Zhizi)	Clearing heat and detoxification	I/R model (30 min/40 min)	SD rats	200 g/kg, 400 g/kg, and 800 mg/kg	7 days	Anti-inflammatory	Increase in the expression of IκBα, and decrease in the expressions of NIK, IKKβ, and NF-κB	Fu et al. (2013a)
Si-Miao-Yong-An decoction (Yuanshen, Jinyinhua, Danghui, and Gancao)	Clearing away heat and toxic matter and activating blood for acesodyne	I/R model (45 min/42 days)	Kunming mice	12 g/kg and 24 g/kg	28 days	Anti-apoptosis, anti-inflammatory and regulating autophagy	Decrease in the expressions of collagen I, MMP9, and TNFα; decrease in the expressions of p-mTOR/mTOR, NLRP3, procaspase 1, and cleaved-caspase 1; and increase in the expression of LC3B-II/LC3B-I	Cui et al. (2021)
		H/R model (4 h/20 h)	H9c2 cell	75 μg/ml and 150 μg/ml	20 h			
		I/R model (45 min/28 days)	SD rats	0.8 g/kg and 1.6 g/kg	28 days	Anti-inflammatory	Decrease in the expressions of NLRP3, ASC, caspase-1, IL-1β, and IL-18	Wang et al. (2022c)
Gualou Xiebai decoction (Gualou, Xiebai, Banxia, Guizhi, Houpu, Zhishi, and Baijiu)	Promoting qi circulation, relieving depression, activating Yang, removing obstruction, and eliminating phlegm	I/R model (30 min/90 min)	SD rats	4 g/kg	6 weeks	Anti-apoptosis	Increase in the expression of ATP5D, and decrease in the expressions of RhoA and ROCK.	Yan et al. (2018)
Huoxue Huatan decoction (Chenpi, Banxian, Baifuling, Gancao, Dafupi, and Zhiqiao)	Activating blood, expelling phlegm, and opening blood vessels	I/R model (40 min/2 h)	Wistar rats	5.02 g/kg, 10.03 g/kg, and 20.06 g/kg	4 and 8 weeks	Regulating mitochondrial energy metabolism and protecting the structure and function of the mitochondria	Increase in the expressions of PGC-1α, PPARα, NRF1, and mtTFA.	Lin et al. (2020)
Sini decoction (Fuzi, Ganjiang, and Gancao)	Warming middle energizer, dispelling cold, and restoring Yang	I/R model (1 h/1 h)	SD rats	5 g/kg	3 days	Anti-oxidation	Increase in the expressions of NF-kB, MnSOD, and Cu-ZnSOD.	Li et al. (2014)
Dang Gui Si Ni decoction (Danggui, Guizhi, Shaoyao, Xixin, Tongcao, Dazao, and Gancao)	Warming channel, expelling cold, and nourishing blood vein	I/R model (30 min/120 min)	SD rats	30 mg/kg	2 h	Anti-oxidation	Decrease in iNOS level, and increase in peNOS and eNOS levels	Qian et al. (2014)
Shexiang Baoxin Pill (Shexiang, Rensheng, Niuhuang, Rougui, Suhexiang, Chansu, and Bingpian)	Supplementing qi and activating blood circulation	I/R model (30 min/4 h)	C57BL/6 mice	20 mg/kg	28 days	Anti-pyroptosis, activating autophagy, and anti-inflammatory	Increase in the expression of mmu-miR-543–3p, and decrease in the expressions of IL-1β, IL-18, mmu_circ_0005874, and Map3k8	Yu et al. (2022)
		H/R model (6 h/18 h)	NRCMS	25 μg/ml	24 h			
Cardiotonic Pill (Danshen, Sanqi, and Bingpian)	Boosting qi and nourishing Yin and strong heart nerves	I/R model (45 min/24 h)	SD rats	10 mg/kg, 20 mg/kg, and 40 mg/kg	7 days	Regulating AA P450 enzyme metabolism	Increase in the expressions of Cyp1b1, Cyp2b1, Cyp2e1, Cyp2j3, Cyp4f6, and EETs, and decrease in the expressions of CYP2J and CYP2C11	Xu et al. (2016)
		H/R mode (6 h/18 h)	H9c2 cell	0.2 mg/ml	24 h			
Shenmai injection (Renshen and Maidong)	Nourishing Yin, generating body fluid, tonifying qi, and preventing exhaustion	I/R model (45 min/24 h)	SD rats	10 μg/ml, 25 μg/ml, and 50 μg/ml	28 days	Anti-apoptosis, reducing mitochondrial fission, and regulating autophagy	Increase in the expressions of Parkin, Beclin 1, LC3BII/I, MFN1, MFN2, and OPA.	Yu et al. (2019)
		H/R model (12 h/2 h)	H9c2 cell	1 μl/ml, 2.5 μl/ml, and 5 μl/ml	24 h			

### 3.1 Single Chinese traditional herbs for treating MIRI

#### 3.1.1 Danshen

*Salvia miltiorrhiza*, also known as “Danshen” in China, was primarily recorded in “Shen Nong Ben Cao Jing.” The Chinese traditional herb “Danshen” refers to the dry roots and rhizomes of *Salvia miltiorrhiza* Bge., which is famous for its effects in promoting blood circulation and removing blood stasis; it has been extensively applied for many years to treat various diseases, especially coronary heart diseases and cerebrovascular diseases, either alone or in combination with other Chinese plant-based medicines (Su et al., 2015). Danshen plays a significant role in protection against cardiovascular diseases through the antioxidant effect (Zhou et al., 2020), anti-inflammatory effect (Wang et al., 2020f), and anti-apoptosis effect (Deng et al., 2021), making investigations on how the effective components of Danshen could be applied to MIRI therapies a hot topic.

To date, more than 100 types of components in Danshen have been identified, among which tanshinone IIA (TA) is present in relatively higher concentrations and is one of the most effective components being applied to CVD treatment. Many studies have provided evidence for the remarkable effect of TA on MIRI. For example, TA has been reported to improve cardiac function by inhibiting collagen I, collagen III, tissue inhibitors of metalloproteinases (TIMP-1), and galectin-3 mRNA expression and enhancing matrix metallopeptidase (MMP)-2 mRNA and protein expression to inhibit myocardial fibrosis and improve myocardial remodeling in rats with I/R-induced heart failure (Zhang et al., 2022). Some studies have also demonstrated that the cardioprotective effect of TA is linked to the JAK2/STAT3 signaling pathway to suppress cell apoptosis (Wang H. et al., 2021). Sodium tanshinone IIA sulfonate (STS), one of the derivatives of TA, has been further developed as an injected medication for treating coronary heart disease, angina, and myocardial infarction in China. STS injection not only improves myocardial function but also regulates the immune response by inhibiting cardiomyocyte apoptosis (Li Y. et al., 2019). STS pretreatment applied to the I/R rat model showed that the downregulated expressions of IL-1β and HMGB1, which mediated the expression of the IL-23/IL-17 axis, reduced neutrophil infiltration, reduced myocardial infarct size, and improved MIRI (Ding et al., 2020a; Ding et al., 2020b). Cryptotanshinone (CTS) inhibited I/R-induced cardiomyocyte apoptosis *in vivo* and *in vitro via* the MAPK pathway (Wang R. et al., 2021). Moreover, CTS is likely to alleviate MIRI by inhibiting the expressions of caspase-12, CHOP, and GRP78 in the myocardial tissue of the I/R rats *via* the activation of the JAK1/STAT3 signaling pathway (Wang R. et al., 2022). Dihydrotanshinone I (DT) and protocatechuic aldehyde (PCA) are also two active ingredients of Danshen against cardiovascular ischemic injury. DT has been found to induce transient ROS generation *via* the reversible inhibition of mitochondrial respiratory complex I and thereby stabilize HIF-1α, which upregulates Nrf2 and activates antioxidant enzymes. PCA can protect cardiomyocytes from reperfusion injury by elevating the levels of reduced glutathione (GSH), which is achieved by providing reducing equivalents to scavenge ROS (Jiang et al., 2019). Furthermore, PCA potently protects cardiomyocytes by suppressing the PERK, IRE1α, and ATF6α signaling pathways, which are involved in regulating endoplasmic reticulum (ER) stress (Wan et al., 2021).

Other components of Danshen have also been found to effectively treat MIRI. Salvianolate is a class of hydrophilic components, among which magnesium lithospermate B is a prominent one and has been shown to improve cardiac function. Salvianolate was reported to significantly inhibit cardiomyocyte apoptosis by regulating apoptosis-related proteins such as Bcl-2, Bax and active caspase-3, indicating the potential therapeutic ability of salvianolate against MIRI (Li M. et al., 2019). Magnesium lithospermate B exhibits the function of resisting myocardial cell programmed necrosis (Wang et al., 2017). Another ingredient called Danshensu (DSS) has been shown to inhibit miR-199a-5p expression. Downregulation of miR-199a-5p mRNA was followed by a rise of phosphorylated Akt (p-Akt) and phosphorylated ERK (p-ERK), implying that miR-199a-5p mRNA plays a role through the PI3K/Akt and ERK1/2 signaling pathways (Shi Y. et al., 2021). A total of 10 μmol/L DSS was able to protect the heart from I/R injury, which was potentially attributed to inhibiting excessive ROS through the Sirt1/FoxO1/Rab7 signaling pathway (Sun et al., 2020). Salvianolic acid A has been shown to protect against MIRI in rats by playing an antiplatelet role and lowering serum levels of TNF-α, IL-1β, and nitric oxide (NO) (Yuan et al., 2017). Sal B attenuates myocardial I/R injury by activating the PI3K/Akt pathway, which was reported to decrease inflammatory responses and inhibit apoptosis in rats (Liu C. et al., 2020).

In addition, the polysaccharides of Danshen have a variety of biological activities such as anti-inflammatory, antioxidant, and immunomodulatory actions (Song et al., 2013). However, reports on the mechanism of action of polysaccharides against MIRI remain rare.

#### 3.1.2 Sanqi

It has been more than 600 years since *Panax notoginseng*, or Sanqi, was first discovered and recorded in ancient China. Sanqi was conventionally used for stopping bleeding, promoting blood circulation, and pain relief, as recorded in “Ben Cao Gang Mu.” Modern studies have suggested that Sanqi is widely used in the prevention and treatment of cardiovascular and cerebrovascular diseases, owing to its significant effects on cerebrovascular dilation, vessel resistance reduction, and vessel endothelial function improvement.

*Panax notoginseng* saponins (PNS) are one of the major active components in Sanqi. It has been reported that PNS can effectively improve MIRI by reducing oxidative stress, reducing calcium overload, suppressing neutrophil activation and adherence, improving metabolism, increasing anti-apoptotic activity, and modifying endothelial functions of blood vessels (Wang W. et al., 2021). MIRI impacts the membrane stability of cardiomyocytes, which increases membrane permeability, thus resulting in the leakage of myocardial enzymes into the serum. A study revealed that the activities of both LDH and CK significantly decreased after PNS treatment in MIRI models, suggesting that the cardioprotective role of PNS is linked to stabilizing cardiomyocyte membranes, thus restoring the impaired membrane permeability due to MIRI (Shi et al., 2018). Liu et al. found that PNS pretreatment on I/R rats for 7 days enhanced mitochondrial autophagosomes in myocardial cells, which was linked to an increased ratio of LC3II/LC3I and upregulated expressions of HIF-1a, BNIP3, Atg5, and Beclin 1 in the rat myocardial tissue (Liu et al., 2019). In addition, PNS can protect cardiomyocytes against hypoxia/reoxygenation (H/R) injury by inhibiting apoptosis *via* FOXO3a/Akt signaling (Liu H. et al., 2021). These cases have shown that PNS could promote autophagy to protect cardiomyocytes from I/R injury, and mitochondrial autophagy has been considered a potential target of PNS in MIRI treatment. Other studies have found that PNS can protect cardiomyocytes by increasing the expression of Mir-30C-5p, which subsequently downregulates p53 expression and inhibits cell apoptosis (Wang et al., 2020g). PNS consists of a variety of monomer components, among which notoginsenoside R1 (NGR1) has been proven to have cardioprotective effects in terms of regulating oxidative stress, endoplasmic reticulum stress, apoptosis, and other relevant signaling pathways (Yu et al., 2016). One study (Zhou and Liu, 2019) demonstrated that NGR1 preconditioning inhibited MIRI by activating the PI3K/Akt signaling pathway, reducing oxidative stress and inflammation, and inhibiting cardiomyocyte apoptosis. NGR1 (20 μM) was likely to alleviate H/R-induced H9c2 cell damage *via* the upregulation of miR-132 and downregulation of its target protein HBEGF (Jin P. et al., 2021).

As a major ingredient of Radix ginseng, ginsenoside Rg1 (Rg1) has been increasingly recognized to benefit the heart. Rg1 (5 mg/kg/h) could prevent myocardial injury caused by I/R, and the mechanism may be related to inhibiting myocardial apoptosis and regulating energy metabolism *via* the RhoA signaling pathway (Li et al., 2018). The compatibility of Sanqi compounds with other herbs is also a hot topic for MIRI treatment. Li et al. found that the anti-inflammatory efficacy of PNS combined with safflower total flavonoids (STFs) was stronger than a formulation containing PNS or safflower extract alone (Li W. et al., 2019). More specifically, PNS and STF together inhibit the activation, aggregation, and adherence of neutrophils by suppressing NF-кB activities or suppressing the combination of platelet-activating factor (PAF) and its receptor in an additive manner (Dong et al., 2014). Wang et al. investigated the therapeutic effect of the compatibility of *Rhizoma coptidis* total alkaloids and PNS on diabetes-induced coronary heart disease using a diabetic MIRI rat model. The combination of components downregulated the expressions of NOX2 and NOX4, which participate in diabetic macroangiopathy and induce ROS synthesis; therefore, the combination treatment inhibited the activation of the NLRP3 inflammasome and other apoptosis-related factors (Wang X. et al., 2021). As most CVD patients are known to develop complications such as diabetes, it is meaningful to explore novel therapies for MIRI patients with different complications.

#### 3.1.3 Huangqi

*Astragalus membranaceus* is a flowering medicinal plant. “Huangqi,” in China, is specifically referred to as the dried root of *Astragalus membranaceus* (Fisch.) Bge. Modern studies have confirmed that astragalus has a series of effects attributed to its rich chemical composition, including neuroprotection, anti-fatigue, renal protection, hepatoprotection, anti-cancer, and cardiac protection (Durazzo et al., 2021). Astragaloside-IV (AS-IV), a marker component for the quality evaluation of Huangqi, has been reported to exhibit cardioprotective properties against MIRI through different pathways, whose underlying mechanisms will be discussed as follows. Studies have shown that AS-IV can significantly alleviate the pathological state of MIRI by regulating autophagy (Shao et al., 2020). In a study by Yin et al. (2019), AS-IV was shown to attenuate MIRI in rats *via* the inhibition of calcium-sensing receptor-mediated apoptotic signaling pathways. Other studies have found that AS-IV could upregulate miR-101a expression, suppress TGFBR1 and TLR2 expressions, and inhibit the MAPK signaling pathway in H/R-injured cardiomyocytes (Wu et al., 2017). Yang et al. (2019) found that AS-IV could regulate the expressions of Nrf2 and Bach1 proteins in the nucleus. AS-IV also promoted the expression of HO-1 (an antioxidant enzyme) to inhibit oxidative stress and inflammation *via* the PI3K-mediated pathway. Li et al. (2017a) reported that AS-IV might inhibit ER stress and prevent the abnormal opening of mPTP through the regulation of GRP78, GRP94, and IRE1. These studies provided evidence for the ability of AS/IV to ameliorate MIRI *via* multiple pathways. Astragalus polysaccharide not only has a recognized therapeutic effect on diabetic complications but also has a notable anti-myocardial ischemia–reperfusion injury. Astragalus polysaccharides (ASPs) are another main active ingredient of Astragalus membranaceus. ASP could reduce intracellular ROS and inhibit apoptosis, and these effects were achieved by the downregulation of the expressions of caspase-3 and Bax and the upregulation of the expression of Bcl-2 both *in vivo* and *in vitro* (Liu D. et al., 2018). Another study has shown that ASP improves myocardial microvascular endothelial cells in H/R by mediating the PI3K/Akt/eNOS signaling pathway (Zhou et al., 2018). Zhao’s team previously showed that the combined action of AS-IV and Rg1 could not only effectively lower blood lipids but also protect against MIRI. Further studies showed that AS-IV and ginsenoside Rg1 could significantly increase the expressions of eNOS and Bcl-2, while decreasing the expressions of Bax and cytochrome C, suggesting that the formulation protects against MIRI mainly through regulating cell apoptosis, potentially *via* the upregulation of the eNOS protein (Zhao T. et al., 2021), yet the study did not analyze the optimal dose ratios of AS-IV and Rg1, which needs further study.

#### 3.1.4 Huangqin

The term “Huangqin” refers to the dried root of *Scutellaria baicalensis* Georgi, which has been involved in medication in China for decades due to its excellent pharmacological properties. Flavonoids are the most abundant components in *Scutellaria*, mainly including baicalin (BA) and hanbaicalin and their aglycones, which are baicalein and wogonin (WG), respectively (Billah et al., 2019). Studies have confirmed that most flavonoids have a certain protective effect on the myocardium, and the bioactive components of Huangqin elicit a cardiac protective effect through some channels (Sun W. et al., 2021). Li and Yu’s (2021) study has shown that WG could increase Nrf2 expression and regulate the redox system balance in MIRI rats (Li et al., 2021). Additionally, the expression of HO-1 also increased. As an HO-1 inhibitor was found to partly reverse the protective effect, it suggested that WG activated Nrf2, induced the expression of the downstream HO-1 gene, and exhibited antioxidant activity (Song et al., 2019). BA, another component of Huangqin, has shown reverse H/R-induced oxidative stress and apoptosis, which was mainly attributed to the enhancement of the activity and expression of aldehyde dehydrogenase (ALDH) in cardiomyocytes (Jiang et al., 2018). Another study has also reported that BA could promote cell survival under oxidative stress by acting on MARCH5 (a ubiquitin ligase of the mitochondrial outer membrane) in cardiomyocytes (Li W. et al., 2020).

The cardioprotective effect of baicalein (an aglycone of BA) is associated with inflammation-related pathways such as signaling through MAPK, Akt, NF-κB, and JAK-STAT (Ma et al., 2018). However, more research will have to be carried out to specify the underlying mechanism of action.

#### 3.1.5 Dahuang

Rhubarb is a kind of heat-clearing and fire-purging drug from *Rheum palmatum L*., *Rheum tanguticum* Maxim. ex Balf., or *Rheum offcinale* Baill*.* and has also been shown to have a protective effect on the heart. Emodin (Emo) is an anthraquinone compound extracted from *Rheum*, the root rhizome of which was primarily used to treat constipation and other gastrointestinal problems. Pharmacological properties of Emo have been demonstrated as anti-inflammation, platelet aggregation inhibition, immune regulation, and anti-tumor (Li et al., 2017b; Gao et al., 2020; Wei et al., 2021). It has been reported that Emo protects against myocardial damage by easing oxidative stress. Other studies have uncovered the effect of Emo as an antioxidant in cardiomyocytes *via* the Nrf2/ARE/HO-1 signaling pathway, which has been proven to show important protective activity against MIRI (Cui et al., 2020).

Other studies have found that Emo can alleviate myocardial I/R injury by inhibiting pyroptosis due to its powerful anti-inflammatory effect, and the TLR4/MyD88/NF-κB/NLRP3 inflammasome pathway is involved in the process (Ye et al., 2019). Rhein, another anthraquinone compound, upregulated p-Akt and activated the Akt/GSK3β/p38 pathway, thereby protecting H9c2 cells from H/R-induced injury (Liu J. et al., 2018).

#### 3.1.6 Shaoyao

Paeonol (Pae) is a bioactive compound derived from the root bark of the Moutan Cortex. Studies have demonstrated the outstanding antioxidant effect of Pae in multiple pathogenetic processes, implying that Pae is a potential option for treating oxidative stress-induced diseases. A study by Choy et al. (2017) reported that Pae increased the utilization of NO in mice and inhibited the production of ROS in the aorta, thereby improving endothelial function and reducing ER stress-mediated oxidative stress. Another recent study also revealed that Pae alleviated MIRI *via* the upregulation of sirtuin-1 (SIRT1) (Liu H. et al., 2020). SIRT1 is a protein that shows inhibitory effects on oxidative stress by regulating Nrf2 and the forkhead box O (FoxO) transcription factors. In addition to its antioxidant properties, SIRT1 also contributes to CVD treatment by reducing the inflammatory response (Hori et al., 2013; Wan et al., 2016; Shah et al., 2017). In the model of MIRI, the expression level of SIRT1 markedly decreased, while adding Pae recovered the expression of SIRT1. Knockdown of SIRT1 decreased the cardioprotective effects of Pae, so it is plausible that Pae at least partly mediates the activity of SIRT1 and potentially affects downstream proteins such as Nfr2 and FoxOs.

An intravenous infusion of Pae 15 min before a ligation operation on rats was found to improve the cardiac function in I/R-injured myocardium and reduce cardiac I/R-induced arrhythmias and mortality (Tsai et al., 2021). Pae inhibits apoptosis and autophagy cell death and alleviates I/R injury by controlling the expressions of apoptotic proteins caspase-8, caspase-9, caspase-3, PARP, Beclin-1, P62, LC3-I, and LC3-II. More investigation of the effects of Pae on the downstream pathway is necessary.

#### 3.1.7 Lianqiao

Hyperoside (Hyp) is a kind of flavonoid compound extracted from many plants, such as *Hypericum perforatum* L. Flavonoid compounds have been proven to contribute to the treatment of CVD (Russo et al., 2019; Parmenter et al., 2020; Gao et al., 2021). Some researchers found that Hyp preconditioning reduced the myocardial infraction area and the apoptotic rate in rats, the mechanism of which was likely to be anti-lipid peroxidation, anti-free radical synthesis, and anti-apoptosis (Xu et al., 2022). Recently, a study revealed the effect of Hyp on H/R-induced cell injury by suppressing Bcl-2-interacting protein 3 (Bnip3) expression (Xiao et al., 2017). Numerous studies have shown that there are large numbers of cytokines associated with the process of H/R, among which Bnip3, as a mitochondrial membrane protein, is a key factor leading to mitochondrial dysfunction and cell damage. Hypericin pretreatment of cardiomyocytes showed that it could inhibit the increase of Bnip3, Bax, and cleaved caspase-3 activity caused by H/R, increase the expression of Bcl-2, and then inhibit cell apoptosis to improve cardiomyocyte injury.

Another study pointed out that Hyp regulated cell proliferation, differentiation, and apoptosis to resist MIRI through PKC/mitoKATP protein kinase C (PKC) (Wang, 2020). Subtypes of PKC include PKCα and PKCε. PKCα has been claimed to be important in the pathological changes during MIRI by reducing Ca^2+^ overload and inhibiting apoptosis (Trerotola et al., 2017; Xu et al., 2019). Wang suggested that the beneficial effect of Hyp on MIRI is associated with the PKC/mitoKATP signaling pathway. Hyp directly upregulates the activities of both PKCα and PKCε, activating downstream mitoKATPC. In this way, Hyp suppresses the accumulation of excessive Ca^2+^ in the cytoplasm, maintains mitochondrial functions, reduces cell apoptosis, and exhibits anti-MIRI actions.

#### 3.1.8 Dengzhanxixin

Breviscapine (Bre) is a flavonoid compound extracted from the plant *Erigeron breviscapus* (vant.) Hand-Mazz. A pharmacological study has shown that Bre not only has beneficial effects on cerebrovascular vessels but also inhibits platelet aggregation and oxygen radicals (Gao et al., 2017; Wen et al., 2021). The study of Wang Y. et al. (2021) emphasized the antioxidant effect of Bre, showing that it promotes the expression of a transcription factor, the cAMP-response element binding protein (CREB), which promotes autophagy and suppresses the expression of toll-like receptor 4 to protect against oxidative damage and apoptosis, thus recusing endothelial cells from MIRI. Additionally, the effect of Bre in rat models of MIRI on diabetes was also studied (Yang N. et al., 2021). mTOR is a vital autophagy-associated factor that is critical for MIRI. The level of mTOR significantly dropped in the sham group, while Bre restored it to normal. Moreover, Bre also increased the levels of phosphorylation of PI3K and Akt and reduced the release of inflammatory factors. Another study reported that the activation of the PI3K/Akt/mTOR signaling pathway exhibited an inhibitory effect on excessive autophagy induced by MIRI (Li et al., 2018), which was consistent with the results of Yang Y. K. et al. (2021). Therefore, through the regulation of the PI3K/Akt/mTOR signaling pathway, Bre suppresses autophagy and the inflammatory response in the myocardium.

#### 3.1.9 Huanglian

Berberine (Ber) is a kind of alkaloid extracted from various kinds of herbs, including *Coptis chinensis* (“Huanglian”). Ber exhibits anti-inflammatory, antibacterial, and anti-tumor activities (Liu and Zhou, 2019). The cardioprotective effect of Ber has also been widely studied. In a study by Sun et al., after Ber treatment in cardiomyocytes *in vivo*, mitochondrial swelling and left ventricle damage declined, and the levels of serum CK-MB and cTnI markedly dropped, indicating that Ber relieved mitochondrial injuries and MIRI. The study also demonstrated that the presence of Ber promoted the expressions of PINK1, P62, LC3B, and Parkin and elevated the expression of USP30 back to its normal level. In conclusion, researchers suggested that Ber promoted mitochondrial autophagy through the activation of the PINK1/Parkin/P62/LC3B autophagy pathway and normalized UP30 expression (Kulek et al., 2020; Sun J. et al., 2021).

#### 3.1.10 Shanzha

Vitexin (VT) is a bioactive flavonoid component of dried leaves from *Crataegus pinnatifida*. According to modern pharmacology, generally, flavonoid compounds extracted from *Crataegus pinnatifida* leaves have certain benefits such as increasing coronary blood flow, preventing myocardial ischemia, and exhibiting antioxidant properties (Fu J. H. et al., 2013; Li et al., 2023). It has been reported that VT exhibits a wide range of pharmaceutical activities, including blood pressure-lowering, anti-inflammation, anti-tumor, and neuroprotective effects (Lee et al., 2012; Demir Özkay and Can, 2013; Yang et al., 2014). More importantly, VT also shows cardioprotective effects, as it has been found to protect against MIRI *in vivo* and H/R injury *in vitro* (Dong et al., 2008; Dong et al., 2011). According to Xue (2020), VT attenuates MIRI through the Epac1–Rap1 pathway. Exchange protein activated by cAMP 1 (Epac1) mainly exists in the heart, kidney, blood vessels, and central nervous system. Rap1 is a downstream GTPase of Epac1. Knockdown of Epac1 was found to prevent MIRI in the laboratory, while agonists of Epac1 worsened myocardial damage and mitochondrial dysfunction. VT preconditioning significantly inhibited the expressions of Epac1 and Rap1, reduced the myocardial infarction area, and eased left ventricle functional failure and mitochondrial failure, indicating that Epac1 signaling plays an important role in the mechanism of VT easing MIRI-induced mitochondrial dysfunction and suppressing the activation of mitochondria-induced apoptosis (Yang H. et al., 2021).

In addition to TCM, the myocardial protective effects of other Chinese herbs have also been confirmed, including antioxidant, anti-inflammatory, immunomodulatory, anti-apoptotic, and other effects to resist MIRI. Examples of these herbs include *Rhodiola rosea* (Sun et al., 2018; Jin Z. et al., 2021; Tian et al., 2022), flowers of *Carthamus tinctorius* (Min and Wei, 2017; Lu et al., 2019), Ginkgo biloba (Liu J. et al., 2020), *Rhizoma acori graminei* (Xiao et al., 2020), and *Schisandra chinensis* (Zhang et al., 2017).

### 3.2 Chinese herbal compound prescription for treating MIRI

#### 3.2.1 Yiqi Huayu decoction

Yiqi Huayu decoction is a classical representation of the established Chinese traditional treatment that “activates blood circulation and removes blood stasis.” The decoction is made of five well-known medicinal herbs, namely, Danshen, Sanqi, Wuzhi Maotao (*Ficus simplicissima* Lour.), Xianhecao (*Agrimonia pilosa* Ledeb.), and Hongjingtian (*Rhodiola crenulata*). In addition to Danshen and Sanqi (which were already discussed earlier), the other three herbs also show outstanding cardioprotective effects against MIRI. Psoralen is the major bioactive component of *Ficus simplicissima* Lour. and contributes to anti-coagulation and immunoregulation. *Agrimonia pilosa* Ledeb. has been proved to show great anti-inflammatory effect. *Rhodiola crenulata* is a well-known herb with an antioxidant property (Huang and Liao, 2012; Miao et al., 2013; Feng et al., 2021; Ye et al., 2021). An *in vivo* study has proven that Yiqi Huayu decoction effectively suppresses the expressions of proteins inducing apoptosis, for instance, Bax, caspase-3, Fas, and P53, and, in contrast, promotes the expression of Bcl-2 (Lin X. et al., 2015). MIRI induces an increase in MDA and a decrease in superoxide dismutase (SOD), which is reversed by Yiqi Huayu decoction (Huang and Liao, 2012; Miao et al., 2013; Lin K. et al., 2015). Additionally, the formulation of the decoction is not fixed. Compared to the formulation described earlier, some people replaced Wuzhi Maotao and Hongjingtian with Tubiechong [the dried body of *Eupolyphaga sinensis* Walker or *Steleophaga plancyi* (Boleny)] and Wuzhaolong (the root or branch of *Ficus hirta* Vahl.), respectively. The new formulation was found to alleviate MIRI in laboratory conditions, which was consistent with clinical observations, and the underlying mechanism was considered to be linked to an increase in SOD activity and a decrease in TNF-α and MMP-9, underpinning the antioxidant and anti-inflammatory effects of Yiqi Huayu decoction (Huang and Liao, 2012).

#### 3.2.2 *Angelica sinensis* decoction

Liu et al. studied *Angelica sinensis* decoction, the effective ingredients of which include paeoniflorin, ferulic acid, glycyrrhizic acid, and cinnamic acid. The effects of *Angelica sinensis* decoction on MIRI-induced oxidative stress in rats and the most optimal concentrations of the components used in an orthogonal test are as follows: the combination that worked best contained ferulic acid, 300 mg/kg; cinnamic acid, 200 mg/kg; and glycyrrhizic acid, 50 mg/kg; which significantly reduced MDA and increased SOD in the myocardium, yet paeoniflorin had a less significant effect on oxidative stress injury (Liu H. H. et al., 2021).

#### 3.2.3 Buyang Huanwu decoction

Song et al. (2020) studied the effects of Buyang Huanwu decoction pretreatment on myocardial enzymes and antioxidant functions in MIRI rats. Compared with the non-treated MIRI model group, serum LDH and CK activities in the Buyang Huanwu decoction group decreased significantly. MDA concentration in the serum of MIRI rats also significantly decreased, while SOD activity and NO concentration increased. The mechanism concluded was: the reduction in lipid peroxidation reactions of cell membranes and the protection of myocardial cells from oxidative stress injury during reperfusion.

#### 3.2.4 Huanglian Jiedu decoction

Liu et al. observed the effect of Huanglian Jiedu decoction on myocardial I/R-induced arrhythmia in rats and explored its mechanism of action. Compared with the I/R group, the morbidity and mortality of ventricular tachycardia (VT) and ventricular fibrillation (VF) in the Huanglian Jiedu decoction group were significantly lower; at the same time, the MDA concentration was significantly decreased, and the SOD activity and NO concentration were significantly increased (Fu X. C. et al., 2013; Liu X. W. et al., 2021).

Furthermore, Shenmai injection (Yu et al., 2019), Si-Miao-Yong-An decoction (Cui et al., 2021; Wang C. et al., 2022), Gualou Xiebai decoction (Yan et al., 2018), Huoxue Huatan decoction (Lin et al., 2020), Si Ni decoction (Li et al., 2014), Dang Gui Si Ni decoction (Qian et al., 2014), Shexiang Baoxin pill (Yu et al., 2022), Cardiotonic pill (Xu et al., 2016), and other Chinese herbal compound prescriptions have been widely used in the clinical treatment of patients with different syndromes, under the guidance of traditional Chinese medicine theory. Due to the large number of components in Chinese herbal compound prescriptions, the mechanisms of their myocardial protective effect are also relatively complex. Current studies have shown that most Chinese herbal compound prescriptions mainly exhibit anti-MIRI effects through anti-inflammatory and anti-oxidation mechanisms and affect other mechanisms in the body.

According to TCM theory, the main pathological mechanisms of MIRI are mainly qi deficiency, blood stasis, and phlegm turbidity. Therefore, herbs and compound prescriptions, with the effects of benefiting qi and nourishing Yin, activating blood stasis, dispelling phlegm, and dispersing nodules, are commonly used in clinical practice to prevent and treat MIRI; good therapeutic effects have been achieved. As research on Chinese herbal medicines and compound drugs continues to deepen, not only will the main active components that exhibit myocardial protective effects be discovered, but also the main mechanisms of myocardial protection, including scavenging of oxygen free radicals, anti-oxidation, inhibition of the inflammatory response, and inhibition of the apoptosis of cardiomyocytes. Whether single herbal medicine, a group formula, or an extracted component of a certain herbal medicine, research on multi-pathway, multi-system, and multi-target effects of Chinese herbal medicines is still insufficient. In addition, there is a gap between animal models and clinical practice, and there are few models of TCM evidence; the application of TCM lacks the guidance of the discriminatory treatment system and cannot reflect the advantages of discriminatory treatment. Therefore, it is important to combine modern medicine and technology with traditional Chinese medicine theories of diagnosis and treatment to explore pathological mechanisms of Chinese medicine in order to prevent MIRI and innovate Chinese medicine prescriptions and medications.

## 4 Conclusion

As reviewed above, it could be concluded that TCM has made great contributions to the treatment of MIRI. Their benefits in promoting blood circulation and removing stasis, clearing heat, and detoxification are key aspects against MIRI. *Salvia miltiorrhiae*, *Panax notoginseng*, and *Scutellaria Baicalensis* have been highlighted for their efficacy in alleviating myocardial injury through anti-oxidative, anti-inflammatory, anti-apoptotic, and autophagy regulatory effects *via* the Notch signaling pathway, the JAK2/STAT3 pathway, the PINK/Parkin pathway, and the PI3K/Akt pathway. TCM formulas have been widely used in the clinical treatment of a variety of CVD by adding and subtracting medicine components to achieve disease prevention.

CVD treatment has been a hot topic worldwide that has drawn great interest for decades. Reperfusion injury is a key pathological process in many cardiac diseases and remains a major problem in the medical field. Though a range of Western chemical drugs have been proposed to potently target the causes of CVD, undoubtedly, some chemical drugs can cause severe side effects, and the uses of some of these drugs are also limited by their high costs. In contrast to chemical drugs, TCM provides novel approaches, targeting MIRI due to their characteristics of multi-component, multi-target, and fewer side effects. In particular, the complex chemical composition of TCM formulas allows a single herb or formula to target multiple signaling pathways, highlighting that the components can improve cardiac functions synergistically. The unique advantages of TCM leave it a great space for development. For example, the additive beneficial effects of the combined treatment of TCM and Western medicine are worth further exploration. The beneficial effects are expected to be more remarkable if the dosages of TCM and chemical drugs are well balanced.

Despite the advantages of TCM in treating MIRI, evaluation of the efficacy and safety of herbs and formulas still lacks a systematic method; therefore, advanced technology for TCM assessment is expected to be developed using a variety of research models. Establishing global criteria for evaluating the performances of TCM contributes to popularizing TCM worldwide. Generally speaking, further research on TCM ingredient pharmacokinetics and pharmacodynamics and the most optimal dosages is necessary to provide a clearer blueprint for future TCM development, encouraging the production of more TCM-based treatments targeting MIRI.
